# Analysis of Potato Physiological and Molecular Adaptation in Response to Different Water and Nitrogen Combined Regimes

**DOI:** 10.3390/plants12081671

**Published:** 2023-04-17

**Authors:** Wenyuan Yan, Junhong Qin, Yinqiao Jian, Jiangang Liu, Chunsong Bian, Liping Jin, Guangcun Li

**Affiliations:** State Key Laboratory of Vegetable Biobreeding/Ministry of Agriculture and Rural Affairs Key Laboratory of Biology and Genetic Improvement of Tuber and Root Crop/Institute of Vegetables and Flowers, Chinese Academy of Agricultural Sciences, Beijing 100081, China

**Keywords:** potato, transcriptome, adaptable mechanism, water and nitrogen interaction

## Abstract

Water and nitrogen are essential for potato growth and development. We aim to understand how potato adapts to changes in soil water and nitrogen content. Potato plant adaptations to changes in soil moisture and nitrogen levels were analyzed at the physiological and transcriptomic levels in four treatment groups: adequate nitrogen under drought, adequate nitrogen under sufficient irrigation, limited nitrogen under drought, and limited nitrogen under sufficient irrigation. Many light-capture pigment complex genes and oxygen release complex genes were differentially expressed in leaves when nitrogen levels were increased under drought conditions, and several genes encoding rate-limiting enzymes in the Calvin–Benson–Bassham cycle were up-regulated; furthermore, leaf stomatal conductance decreased, whereas the saturated vapor pressure difference and relative chlorophyll content in the chloroplasts increased. *StSP6A*, a key gene in potato tuber formation, was down-regulated in response to increased nitrogen application, and the stolon growth time was prolonged. Genes related to root nitrogen metabolism were highly expressed, and protein content in the tuber increased. Weighted gene co-expression network analysis (WGCNA) revealed 32 gene expression modules that responded to changes in water and nitrogen levels. A total of 34 key candidate genes were identified, and a preliminary molecular model of potato responses to alterations in soil water and nitrogen content was constructed.

## 1. Introduction

Nitrogen is an essential nutrient for potato growth and development. The nitrogen fertilizer urea is commonly used in agriculture. Urea is hydrolyzed by soil urease to ammonium ions and bicarbonate plasma, which are then absorbed by plant roots. Ammonium in the soil can also be converted to nitrate by microorganisms through nitrification [1]. Nitrates and ammonium salts can both be absorbed and utilized by plants. Proteins in the nitrate transporter (NRT) and ammonium transporter (AMT) families are involved in the uptake and transport of nitrate and ammonium, respectively [2]. The plant enzyme glutamine synthetase converts NH_4_^+^ to glutamine, from which α-ketoglutarate forms amino acids and amides. Increased ammonium levels provide the raw materials for amino acid synthesis; in response, plants up-regulate amino acid transporters to increase amino acid translocation [3]. Plants can also convert nitrate to ammonium salts. Under aerobic conditions, nitrate reductase converts nitrate absorbed by the roots to nitrite, which is then converted to ammonium by nitrite reductase [4]. Excess levels of nitrite and ammonium are toxic to plants. Excessive application of nitrogen fertilizer would destroy the carbon and nitrogen balance, disrupt intracellular pH balance, and affect the oxygen-evolving complex and cations [5]. Therefore, plants must monitor external nitrogen levels and integrate information regarding the nitrogen requirements of different tissues to regulate nitrogen uptake and translocation [6].

Nitrogen is not only a nutrient but an important signaling molecule. Ammonium supply increases cellular levels of reactive oxygen species (ROS), which can act as secondary signals to initiate a variety of signaling events. Ammonium can also activate other enzymes, such as the ROS-scavenging enzymes catalase and glutathione reductase [7]. Cellular nitrogen levels also impact hormone metabolism. Ammonium can interact with genes related to growth hormone pathways, causing changes in the morphological phenotypes of roots and other tissues, and furthermore activate defense responses mediated by abscisic acid (ABA), jasmonic acid (JA), and putrescine to enhance plant resistance [8,9].

Water is essential for the absorption and utilization of nitrogen fertilizer in potato plants, which are often threatened by water deficits. Drought can damage plant cell membrane systems and organelle structures, affecting normal metabolic responses and causing a range of problems, such as enhanced respiration and reduced photosynthesis [10]. Plants respond to drought stress through regulatory mechanisms at multiple levels: morphological (e.g., stomatal movement), physiological (e.g., photosynthetic rate), and molecular (e.g., cellular osmoregulation and synthesis of transport and antioxidant proteins). A variety of secondary messengers (e.g., Ca^2+^, ROS, phosphoglycerol, ABA, and NH_4_^+^) and transcription factors (TFs) play important regulatory roles in drought signaling and adaptation after specialized sensors on the cell membrane detect drought stimuli [11].

While sufficient nitrogen supply can alleviate the negative effects of drought stress by improving the antioxidant enzyme system and maintaining higher osmoprotectants contents, overuse of nitrogen fertilizer will reduce root water absorption and result in greater yield losses induced by the deficit irrigation regime [12]. Plants have several strategies for responding to changes in soil water and nitrogen content. Physiological responses to changes in water and nitrogen levels have previously been analyzed in our laboratory by measuring potato plant indexes under nine treatment conditions with varied water and nitrogen levels [13]. To further understand how potato adapts to changes in soil water and nitrogen content at the transcriptional level, the present study includes analysis and integration of water and nitrogen metabolic regulatory networks in potato by comparing phenotypes and transcriptomes of potato plants in response to four water and nitrogen treatments. The results provide a theoretical reference for water and nitrogen management and the improvement of water and nitrogen use efficiency in potatoes.

## 2. Results and Discussions

### 2.1. Sequencing Data Analysis and Validation

Analysis of the quantity and quality of the transcriptome sequencing data showed that there were 4 × 10^7^ clean reads, corresponding to 6 Gb of sequencing data per sample. The Q30 ratio was ≥ 94% for each treatment group. These results indicated that the sequencing data from all samples were of high quality and met the standards for use in subsequent analyses (Supplementary Table S2). To verify the transcriptomic data, 20 differentially expressed genes (DEGs) were randomly selected from different treatment groups for validation via RT-qPCR. The RT-qPCR results were consistent with the transcriptomic data (R^2^ = 0.8235), indicating that the sequencing results were reliable (Supplementary Figure S1).

### 2.2. Tissue-Specific Variation in Water- and Nitrogen-Responsive Genes

**Figure 1:**
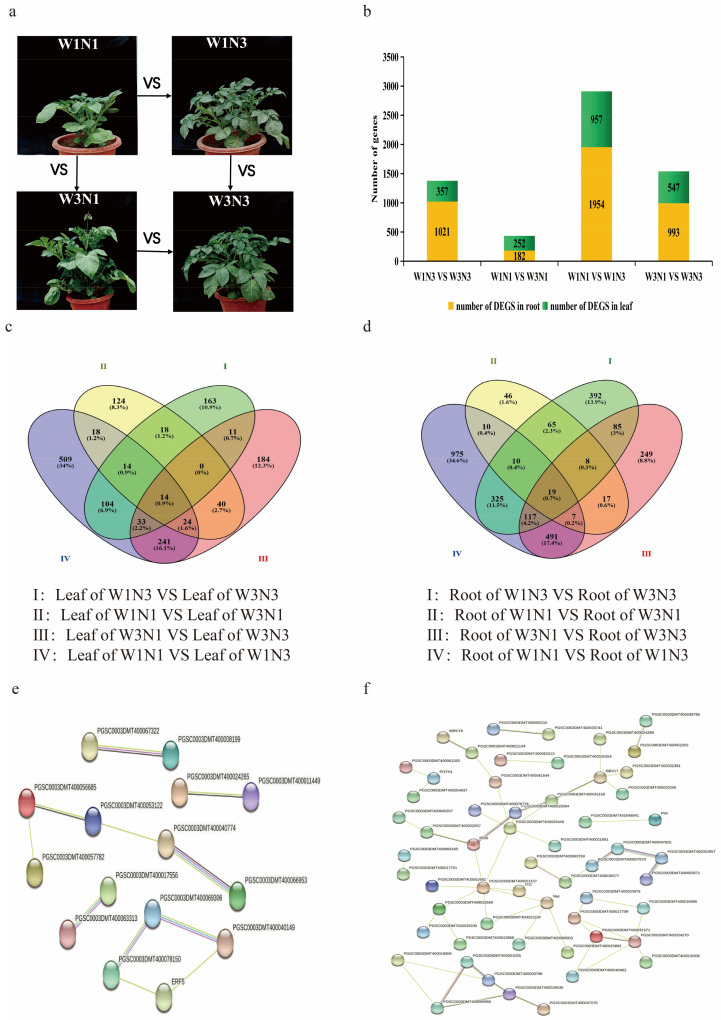
Differentially expressed gene (DEG) identification. (**a**) Diagram showing the samples compared in this analysis. (**b**) Distribution of DEGs (FDR < 0.05 and |log2(fold change)| ≥ 1) between treatment groups in two tissues. (c-d) Unique and shared DEGs between treatment groups in leaf tissue (**c**) and root tissue (**d**). (**e**,**f**) Unique and shared differentially expressed transcription factors (TFs) in leaf tissue (**e**) and root tissue (**f**). In the network, the circular node with a specific number represents the protein, and the interconnecting lines represent the source by which protein interactions are derived. Sources of protein interaction were represented with black, pink, green, and blue lines that represent co-expression, experimental data, text mining, and homology, respectively.

Gene expression was analyzed in potato root and leaf tissues using pairwise comparisons of treatment groups, namely W1N3 vs. W3N3; W1N1 vs. W3N1; W3N1 vs. W3N3; and W1N1 vs. W1N3. A total of eight sets of DEGs were identified from the four treatment group comparisons in two tissues (Figure 1a). The W1N1 vs. W1N3 comparison showed the largest number of DEGs. In both leaves and roots, there were more DEGs between treatments with variations in nitrogen levels than between treatments with variations in water levels (Figure 1b). A crossover analysis of DEGs revealed that there were 14 DEGs in leaves that responded to changes in both water and nitrogen (Figure 1c, Supplementary Table S3); in roots, that number increased to 19 DEGs (Figure 1d, Supplementary Table S4). In potato leaves and roots, there were 693 and 1224 DEGs, respectively, between nitrogen treatments and 287 and 438 DEGs, respectively, between water treatments. These data indicated that there were more DEGs in response to changes in nitrogen levels than water levels and that there was an interaction between responses to water and nitrogen. The common DEGs (14 DEGs in leaves, 19 DEGs in roots) may, therefore, mediate the interaction. TFs in the DEG sets were analyzed. These TFs were found to have a mutual regulatory relationship. The connectivity was higher for TF regulatory networks in potato roots than in leaves, although there was some crosstalk between the regulatory networks in the two tissues. *MIKC_MADS* (PGSC0003DMT400063312) was differentially expressed in both roots and leaves in response to increased nitrogen (W1N1 vs. W1N3), indicating that *MIKC_MADS* played an important role in nitrogen regulation in at least two tissues (Figure 1e,f).

To further understand the functions of DEGs, GO enrichment analysis was performed (Supplementary Figure S2). The results suggested that higher levels of water or nitrogen promoted root cell differentiation and proliferation, hormone and specialized metabolite biosynthesis, and accumulation of photosynthetic products in transit. In leaves, genes that were up-regulated in response to higher water or nitrogen levels were related to chlorophyll and specialized metabolite synthesis, photosynthetic product production and transport, and assimilation and transport of ions and organic matter from the soil. In roots, DEGs between nitrogen treatments were enriched in functions related to nitrogen absorption and assimilation, symbiotic fungi collaboration, miRNA-mediated gene silencing, and other biological processes. Genes that responded to changes in water levels in the roots were enriched in functions related to chromatin morphological changes, histone modifications, symbiotic fungal collaboration, and other biological processes.

### 2.3. Potato Stress Response Was Increased under Drought and Nitrogen Deficit

**Figure 2:**
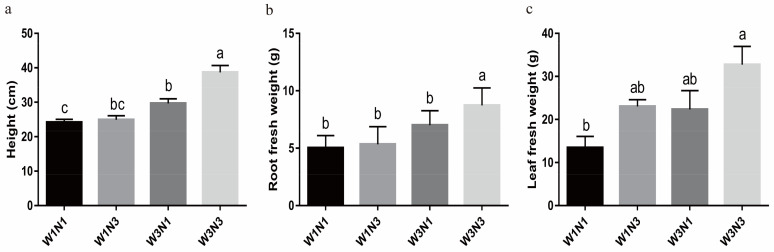
Plant growth indexes under varied water and nitrogen treatments. (**a**) Plant height of each treatment group. (**b**,**c**) Root fresh weight (**b**) and leaf fresh weight (**c**) of each treatment group. Values on bar are mean ± standard error (*n* = 9). Different letters indicate significant differences (*p* < 0.05).

KEGG enrichment analysis was next conducted for DEGs between treatment groups. In response to increased nitrogen under adequate water conditions (W3N1 vs. W3N3) or increased water under adequate nitrogen conditions (W1N3 vs. W3N3), DEGs in both roots and leaves were primarily enriched in cell proliferation, gluconeogenesis, and other metabolic pathways related to cell growth and development (Supplementary Figure S3). Plant height and root fresh weight were also significantly higher after increasing nitrogen levels under sufficient water conditions (W3N1 vs. W3N3) and after increasing water levels under sufficient nitrogen conditions (W1N3 vs. W3N3). There were no significant changes in the fresh weight of either roots or leaves in response to variations in nitrogen or water levels, while there was still a deficit in either input (W1N1 vs. W1N3, W1N1 vs. W3N1) (Figure 2).

Water-responsive genes in root tissue under sufficient nitrogen conditions were enriched in cell replication and proliferation, photosynthetic product metabolism, and photoassimilate accumulation; in leaf tissue, they were enriched in photosynthesis and amino acid metabolism pathways. In addition, root fresh weight and plant height increased in response to additional water under sufficient nitrogen conditions. DEGs in both root and leaf tissues were significantly enriched in pathways related to specialized metabolism and signal transduction. Moreover, plant height and fresh weight were lower under either water- or nitrogen-deficient conditions. This indicates that potato plants enhanced stress response through up-regulation of specialized metabolism, signal transduction, and glutathione metabolism to adapt to low water or nitrogen conditions.

### 2.4. Changes in Soil Nitrogen or Moisture Levels Led to Induction of Distinct Metabolic Pathways

#### 2.4.1. Increased Nitrogen Application under Drought Conditions Can Improve the Efficiency of Light Energy Utilization in Leaves

**Figure 3:**
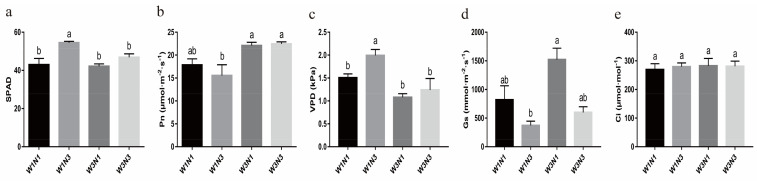
Leaf physiological characteristics associated with photosynthesis. (**a**–**e**) The relative chlorophyll content (**a**), net photosynthetic rate (**b**), saturated vapor pressure difference (**c**), stomatal conductance (**d**), and intercellular CO_2_ concentration (**e**) of each treatment group. Values on bar are mean ± standard error (*n* = 9). Different letters indicate significant differences (*p* < 0.05).

Analysis of genes related to photosynthetic metabolic pathways revealed that 79 genes involved in the light reactions were induced by increased nitrogen levels under drought conditions (W1N1 vs. W1N3). Many genes encoding components of the photopigment protein complex and the oxygen release complex were up-regulated. For example, the oxygen-evolving enhancer protein 2-1 gene (PGSC0003DMG400031395) had a log_2_FC value of 4.5 in this comparison; four genes encoding electron carriers were up-regulated, with one (PGSC0003DMG400004532) having a log_2_FC value of 4.2. Sixteen up-regulated genes were identified as components of the dark reactions, including two key rate-limiting fructose-1,6-2 phosphatase genes (PGSC0003DMG400019189 and PGSC0003DMG400024109) with log_2_FC values of 2.2 and 1.4, respectively. Some members of the Calvin–Benson–Bassham (CBB) cycle were also up-regulated, namely three glyceraldehyde 3-phosphate dehydrogenase genes and several ribulose-1,5-diphosphate carboxylase genes. Thirteen DEGs were associated with photorespiration, most of which were up-regulated (Supplementary Table S5). No significant changes in photosynthetic characteristics were identified when water levels were increased under limited nitrogen conditions. Measurements of physiological photosynthetic characteristics showed that the saturated vapor pressure difference and relative chlorophyll content increased in potato leaves in response to nitrogen addition under drought conditions, whereas the intercellular CO_2_ concentration, net photosynthetic rate, and stomatal conductance remained unchanged. The relative chlorophyll content and saturated water vapor pressure difference decreased, and the net photosynthetic rate increased in potato leaves when water levels were increased under sufficient nitrogen conditions (Figure 3).

#### 2.4.2. Nitrogen Levels Could Affect Expression of Genes Related to Stress Resistance

In response to increased nitrogen levels under drought conditions (W1N1 vs. W1N3), there were 103 hormone-related and 182 cell wall construction-related DEGs in root tissues, such as genes encoding indoleacetic acid (IAA)-amino acid hydrolase 4, ethylene-responsive transcriptional coactivator, jasmonate o-methyltransferase, gibberellin 2-oxidase 1, glycosyl hydrolase family, glucan protein synthase, and β-1,3 glucan hydrolase. Many stress-related genes were also differentially expressed, including those encoding members of the heat shock protein family, oxidoreductase, and ubiquitination-related enzymes. In response to increased water under limited nitrogen conditions (W1N1 vs. W3N1), DEGs included 15 hormone-related genes, four cell wall construction-related genes, nine specialized metabolism-related genes, and several stress-responsive genes that were regulated in root tissues (Supplementary Table S6).

These results indicated that in addition to providing bioavailable nitrogen, urea can act as a signal that interacts with genes related to hormone pathways and specialized metabolism. Further analysis revealed that expression of genes related to ABA synthesis could only be detected in the root, not in the leaf. This suggested a unique regulatory role of roots in response to changes in water and nitrogen availability in the soil. *ATHVA22C* (PGSC0003DMG402023061), a member of the ABA pathway, was up-regulated in root tissue after nitrogen application under normal water or drought conditions (W1N1 vs. W1N3, log_2_FC = 2.17; W3N1 vs. W3N3, log_2_FC = 1.98), indicating that this gene might play a key role in the interaction between nitrogen and ABA.

#### 2.4.3. Nitrogen Transporters Exhibited Tissue-Specific Expression in Potato

Genes related to the nitrogen metabolism pathway were next identified and analyzed. Four high-affinity nitrate transporter genes were identified in root tissues. One of these (PGSC0003DMG400019674) was up-regulated in response to increased water or nitrogen (log_2_FC > 2.0), peaking at log_2_FC = 4.9 in response to increased nitrogen under standard water conditions (W3N1 vs. W3N3). Another high-affinity nitrate transporter (PGSC0003DMG401011998) and three ammonium transporter genes (PGSC0003DMG400046020, PGSC0003DMG401015894, and PGSC0003DMG400028710) were up-regulated in the roots in response to increased water but were down-regulated in response to increased nitrogen. The gene encoding ferredoxin–nitrite reductase (PGSC0003DMG400008262) was down-regulated in response to increased water and up-regulated in response to increased nitrogen.

The gene encoding glutamine synthetase cytosolic isozyme 1-1 (PGSC0003DMG400023620) was up-regulated in leaves in response to increased nitrogen but down-regulated in response to increased water levels. Three ammonium transporter genes were identified as DEGs in leaves, one of which (ammonium transporter 1 member 2 (PGSC0003DMG400028710) was up-regulated in response to increased water levels and down-regulated in response to increased nitrogen (Supplementary Table S7). Tuber protein content was significantly higher under both adequate water and drought when nitrogen levels were increased (Supplementary Figure S4).

In leaves, higher nitrogen levels led to up-regulation of the gene encoding glutamine synthetase cytosolic isozyme 1-1 to promote nitrogen assimilation and utilization. Together, these changes resulted in higher protein accumulation in tubers. High-affinity nitrate transporter 2.4-like genes were specifically expressed in roots, whereas ammonium transporter genes (PGSC0003DMG401015894 and PGSC0003DMG400028710) were expressed in both roots and leaves. These findings indicate that nitrogen uptake and utilization were mainly regulated by nitrate and ammonia transporters and that the nitrate transporter was specifically expressed in the root, whereas the ammonia transporter acted in both roots and leaves.

#### 2.4.4. Increased Nitrogen Fertilization Can Delay Potato Tuber Expansion

**Figure 4:**
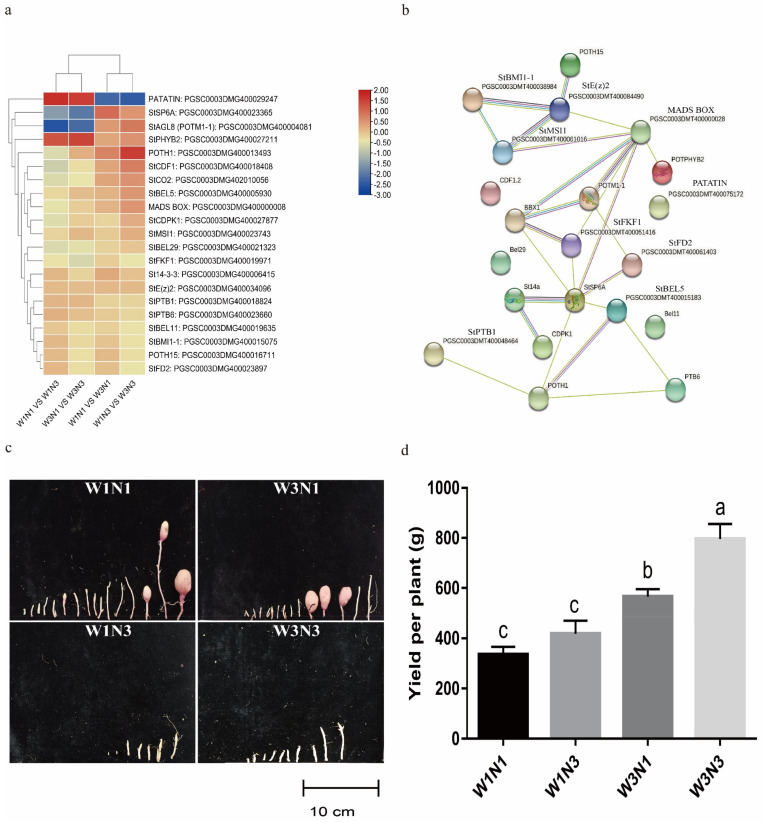
Potato tuber development in response to variations in water and nitrogen levels (**a**) Heatmap showing expression of tuber development-related genes in response to changes in water and nitrogen levels. (**b**) Protein interaction network. In the network, the circular node with a specific number represents the protein, and the interconnecting lines represent the source by which protein interactions are derived. Sources of protein interaction were represented with black, pink, green, and blue lines that represent co-expression, experimental data, text mining, and homology, respectively. (**c**) Tuber development during tuber expansion in each treatment group. (**d**) Yield of each treatment group. Values on bar are mean ± standard error (*n* = 9). Different letters indicate significant differences (*p* < 0.05).

Potato tuber expansion is regulated by leaves. Genes such as *StSP6A*, *StaGL8*, and *StPHYB2* are expressed in leaves, and the corresponding protein products can be transmitted to the stolon to cause stolon sub-apical expansion. Here, we measured the expression of the genes involved in mediating tuber expansion in leaves. Significant differential expression of *PATATIN*, *StSP6A*, *StPHYB2*, and *StaGL8* was observed. Specifically, *PATATIN* was up-regulated in response to increased nitrogen application and down-regulated in response to increased water levels. *StSP6A* and *StaGL8* were down-regulated in response to increased nitrogen and up-regulated in response to increased water. Other regulatory genes were also found to be differentially expressed (Figure 4a, Supplementary Table S8).

Further analysis of DEGs related to tuber expansion revealed that they could interact with each other. *StSP6A*, *MADS BOX*, and *StE(z)2* were hub genes (Figure 4b). Increased nitrogen levels led to the down-regulation of *StaGL8* and the up-regulation of *PATATIN* and *StPHYB2*, eventually leading to the down-regulation of *StSP6A*. Increased water levels led to the down-regulation of *PATATIN* and the up-regulation of *StaGL8* and *StPHYB2*, which then led to the up-regulation of *StSP6A* and the promotion of stolon end extension. Clustering analysis of the gene expression profiles revealed that genes related to tuber expansion clustered together (i.e., showed similar expression patterns) in response to increased nitrogen and that genes responding to increased water levels clustered into another group. Observations of potato stolon developmental morphology showed that changes in soil nitrogen content did affect stolon development. The stolon entered the tuber expansion stage in nitrogen-deficient conditions (W1N1 and W3N1) but was in the sub-apical expansion stage under sufficient nitrogen conditions (W1N3 and W3N3) (Figure 4c). There was no difference in per-plant yield when nitrogen levels were increased under drought conditions, but there was a significant increase in yield when nitrogen levels were increased under sufficient water conditions or when water levels were increased (Figure 4d). Thus, nitrogen limitation promoted tuber expansion and accelerated entry of stolons into the tuber expansion phase; however, this failed to increase yield. In contrast, sufficient nitrogen treatment prolonged the period of stolon development.

### 2.5. Screening of Key Genes Responding to Changes in Water and Nitrogen Levels

**Figure 5:**
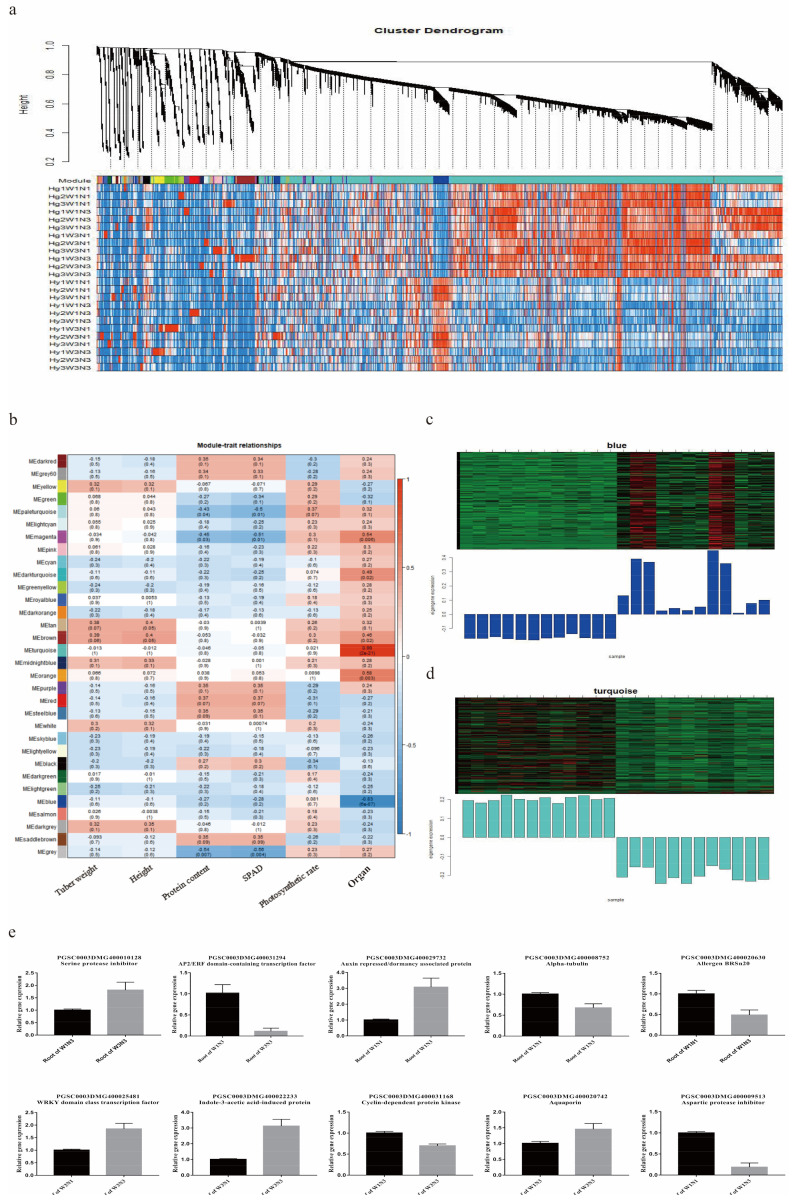
Key candidate water- and nitrogen-responsive genes screened with weighted gene co-expression network analysis (WGCNA). (**a**) Hierarchical cluster tree showing co-expression modules identified by WGCNA. Upper, gene cluster tree; each branch corresponds to a module. Lower, gene heatmap; rows represent all genes in a module, and columns represent samples. (**b**) Module–trait associations from WCGNA. For each cell, the upper number is the Pearson correlation coefficient, and the lower number is the significance value. (**c**) Upper, expression of each gene in the blue module in each sample. Each row represents all genes in the module, and each column represents a sample. Lower, expression level of the blue module in each sample. (**d**) Upper, gene expression of each gene in the turquoise module in each sample. Each row represents all genes in the module, and each column represents a sample. Lower, expression level of the turquoise module in each sample. (**e**) Ten candidate water- and nitrogen-responsive genes were selected randomly for RT-PCR validation.

A total of 32 modules were obtained from WGCNA (Figure 5a). Tuber weight, plant height, and other indicators were significantly associated with these modules (Figure 5b). The 14,458 genes in the turquoise module were significantly positively correlated with root tissue (r = 0.99; *p* = 2 × 10^−21^). The 3352 genes in the blue module were significantly positively correlated with leaf tissue and were specifically expressed under limited nitrogen conditions (r = −0.83; *p* = 6 × 10^−7^) (Figure 5c,d). The blue module was significantly enriched in polysaccharide, amino acid, and specialized metabolite metabolic pathways; the turquoise module was significantly enriched in ubiquitination and protein degradation pathways (Supplementary Figure S5). Protein interaction networks were drawn for the genes present in those two modules. Based on their expression levels (and a connectivity threshold of > 0.5), 34 candidate water- and nitrogen-responsive genes were selected (Supplementary Table S9, Figure S6). Ten were selected at random for RT-PCR validation (Figure 5e). The expression levels of these genes differed between the corresponding treatment and control groups, indicating that the 34 candidate genes played important roles in responding to changes in water and nitrogen levels.

## 3. Materials and Methods

### 3.1. Experimental Location

Experiments were conducted from May to September 2020 at the Chabei Experimental Base, Zhangjiakou City, Hebei Province, China. The Chabei Experimental Base (41°15′ N, 114°7′ E) is located at the southern edge of the Inner Mongolia Plateau. It has a mid-temperate continental monsoon climate with an annual average temperature of 3.2 °C, annual average precipitation of ~300 mm, and an annual average of 2897.8 sunshine hours. The minimum and maximum temperatures ranged from 10.05 °C to 21.66 °C over the growing period, and the average daylight time was 8.54 h. The nitrogen-efficient potato variety ‘Zhongshu 28’ was used, which has a fertility period of ~98 d.

### 3.2. Experimental Treatments

Potato tubers were planted in pots (28.5 cm × 24 cm × 26.5 cm) filled with substrate (2:2:1 coir/charcoal/soil). The primary treatment factor was the water regimen: drought (W1) or adequate irrigation (W3). The secondary treatment factor was the amount of nitrogen applied: limited nitrogen (N1) or sufficient nitrogen levels (N3). There were four treatment groups (W1N1, W1N3, W3N1, W3N3). There were 10 pots per replicate and three independent replicates for each treatment. The relative water content of the soil and the amount of nitrogen applied for each treatment are shown in Table 1. Urea (N ≥ 46%), calcium superphosphate (P_2_O_5_ ≥ 12%), and potassium sulfate (K_2_O ≥ 50%) were used as fertilizers, with 10.3 g and 20.47 g of potassium and phosphorus fertilizer, respectively, applied to each pot. For the N1 and N3 nitrogen treatments, 2.24 g and 11.11 g nitrogen, respectively, were applied per pot. Potassium fertilizer was applied in phases, with 40% applied initially and the remainder applied at the seedling (15%), tuber formation (20%), and tuber expansion (25%) stages. All phosphorus fertilizer was applied initially as base fertilizer. Seeds were sown on 10 May, and seedlings emerged on 5 June. One week after seedlings emerged, the soil water content was monitored using the L99-TWS-2 soil temperature and humidity automatic recorder (Hangzhou Luger Technology Co., Ltd.), and additional water was added at the same time each day. Other plant management was conducted in accordance with standard procedures. Plants were harvested on September 13.

### 3.3. Measurement and Analysis of Physiological Indicators

Physiological indicators were measured during the tuber formation stage. Photosynthetic characteristics were measured in the fourth expanded leaf from the top of each potato plant with a CIRAS-3 portable photosynthetic apparatus from 9:00 to 12:00 on a sunny day. Potato plant heights were determined with a measuring tape. Relative chlorophyll content was measured with a PhotosynQ chlorophyll analyzer (PhotosynQ, East Lansing, MI, USA). Three potato plants were randomly selected from each treatment group per replicate to determine the fresh weight of plant organs. During the potato starch accumulation period, three plants were randomly selected from each treatment group per replicate to measure tuber weight, tuber starch content (with the iodine colorimetric method), and crude protein content (with the Kjeldahl method). Microsoft Excel 2016 and SPSS 21.0 were used for data processing and graphing, and analysis of variance (ANOVA) was performed with post-hoc Duncan’s multiple range test to determine significant differences between treatment groups (*p* < 0.05).

### 3.4. RNA-Seq Experiments

The fourth expanded leaves and young roots of plants in each treatment group were sampled at the tuber formation stage. Collected samples were frozen immediately in liquid nitrogen and stored at −80 °C until RNA extraction. Samples were ground in liquid nitrogen. Total RNA was extracted using TRIzol reagent (Invitrogen, Carlsbad, CA, USA). Samples were then treated with TURBO DNase I (Ambion, Austin, TX, USA) for 30 min and purified using the RNeasy^®^ Plant MiniKit (QIAGEN, Hilden, Germany). RNA sequencing libraries were prepared with the TruSeq RNA sample Prep V2 kit (Illumina, San Diego, CA, USA) following the manufacturer’s instructions. The quality and size of cDNA libraries were analyzed using the Agilent 2200 TapeStation system (Agilent, Santa Clara, CA, USA) prior to sequencing. cDNA libraries were sequenced using the Illumina NovaSeq 6000 sequencing system with the 150-cycle paired-end sequencing protocol (Annoroad, China). There were three biological replicates per treatment group.

### 3.5. Transcriptomic Data Analysis

The potato reference genome (http://solanaceae.plantbiology.msu.edu/pgsc_download.shtm/PGSC_DM_v4.03) (accessed on 11 February 2023) and corresponding annotations (http://solanaceae.plantbiology.msu.edu/data/PGSC_DM_V403_genes.gf.zip) (accessed on 11 February 2023) were used for genome alignment and annotation. Raw reads were cleaned and filtered with Fastp, and samples were compared with Hisat2 [14,15]. Gene counts and abundance (in fragments per kilobase of transcript per million mapped reads (FPKM)) were calculated with Stringtie [16]. Differentially expressed genes (DEGs) between treatment groups were identified with the R package ‘Deseq2’ using thresholds of |log_2_(fold change (FC))| ≥ 1 and false discovery rate (FDR) < 0.05 [17]. The R package ‘WGCNA’ was used for weighted gene co-expression network construction and module partitioning. The dynamicTreeCut algorithm was used to perform the hierarchical clustering and divide modules. The gene significance and module membership were calculated to relate modules to traits. The Pearson correlation coefficient greater than 0.8 and *p* < 0.05 were used as thresholds to select the key module. We defined the genes in the key module with a combined score over 0.5 as hub genes. The hub genes in the pathway related to water and nitrogen metabolism with the largest variation selected by the gene expression level were defined as key genes [18]. AgriGO (http://systemsbiology.cau.edu.cn/agriGOv2/#) (accessed on 11 February 2023) was used for Gene Ontology (GO) term enrichment analysis, and the Kyoto Encyclopedia of Genes and Genomes (KEGG) (https://www.kegg.jp/) (accessed on 11 February 2023) was used for biochemical pathway enrichment analysis. Protein–protein interaction (PPI) networks were analyzed with STRING (https://cn.string-db.org/) (accessed on 11 February 2023), using a medium combined score threshold of 0.4. Metabolic pathway analysis was conducted with Mapman [19]. All data used in this study were deposited at the National Center for Biotechnology Information (NCBI) under the project name PRJNA863742.

### 3.6. Validation of Candidate Genes Via Reverse Transcription Quantitative PCR (RT-qPCR)

Primers were designed for candidate genes using Primer 5 (Supplementary Table S1). Reverse transcription reactions were conducted using 2 µg of total RNA and TaqMan Reverse Transcription Reagent (Applied Biosystems, NJ, USA). Each 20 µL reaction system contained 10 µL of Premix Ex Taq (RR420A, Takara, Japan), 0.5 µL each forward and reverse primer (10 μmol/L), 7 µL of double distilled water, and 2 µL of cDNA. The thermocycling program consisted of a denaturation step at 95 °C for 30 s, then 40 cycles of 95 °C for 10 s and 60 °C for 20 s. The relative expression level of each gene was calculated using the 2^−∆∆Ct^ method [20], with *EF-1α* serving as the internal reference gene. All RT-qPCR reactions were performed in technical triplicate.4. Discussion

Nitrogen is crucial for plant growth and development. There are a variety of nitrogen compounds found in nature. The primary forms used by plants are nitrate and ammonium salts, which can be absorbed and transported through transporters specific to those compounds [21,22]. Transporters not only mediate nitrogen sensing and uptake but participate in nitrogen translocation and unloading, and they have different spatial expression patterns [23]. In this study, transporter genes such as *AMT*s, *NRT*s, and aquaporin were identified as differentially expressed in response to changes in water or nitrogen levels. Increased soil levels of water and nitrogen induced three different *AMT* genes (sot: 102585044, sot: 102593437, and sot: 102578704), five *NRT* genes (PGSC0003DMG400011693, sot: 102581584, sot: 102584553, sot: 102588458, and sot: 102596700), and one aquaporin gene (sot: 102589703). Increased nitrogen supply under sufficient water conditions (W3N1 vs. W3N3) also induced an *AMT* gene (sot: 102601799) and an *NRT* gene (sot: 102581584), specifically in the roots, increasing nitrogen uptake. The same *AMT* gene (sot: 102601799) and an ABC transporter protein gene (PGSC0003DMG400033041) were specifically expressed in roots after water levels were increased under limited nitrogen conditions (W1N1 vs. W3N1). These transporters thus play important roles in water or nitrogen uptake and transport (and in stress response). A crossover analysis of DEGs revealed that the *NRT* gene (PGSC0003DMG400011693) and aquaporin gene (sot: 102589703) were responsive to changes in both water and nitrogen. These two genes may, therefore, mediate the interaction between water and nitrogen. Coordination of nitrogen allocation among different tissues is critical for efficient nitrogen utilization in a plant; balancing nitrogen demand and supply requires communication between different tissues. Three *AMT* genes were differentially expressed in leaf tissues, two of which (sot: 102578704 and sot: 102593437) were also differentially expressed in roots. These two *AMT* genes may therefore mediate within-plant ammonium transport. After nitrogen is transported to branches, a plant will either store or assimilate it depending on the developmental stage, nitrogen demand, and leaf capacity. A nitrogen starvation signal from the roots must be enhanced by a local above-ground nitrogen starvation signal to trigger a response [24].

**Figure 6:**
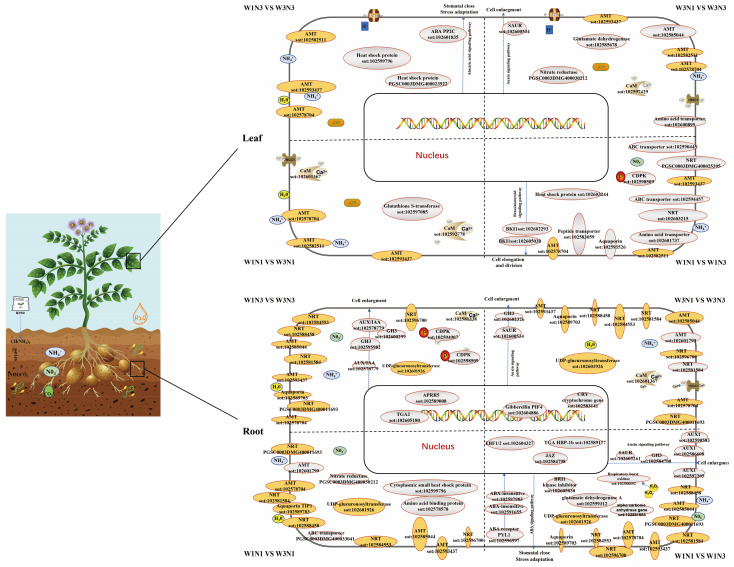
Molecular model of potato responses to changes in water and nitrogen levels.

Plant tolerance to ammonium is related to its capacity for ammonium assimilation [25]. After ammonium and nitrate are transported into cells, they are assimilated through the actions of nitrate reductase and glutamine synthetase [26]. In this study, changes in water and nitrogen levels were found to differentially regulate some nitrogen metabolism-related enzymes, such as glutamate dehydrogenase and nitrate reductase. Ammonia can be used not only as a nitrogen source but also as a signaling molecule to regulate many biological processes, including root morphology, branch development, seed germination, and flowering [27], and ammonia transporters can function not only in transport but in signal transduction [28]. Here, differential expression was detected in genes encoding calmodulin, calcium-dependent protein kinase (CDPK), MAPK, and other signal transduction genes. When ammonium ions in the soil are recognized by ammonium transporters in the roots, the ions can act as signaling molecules to trigger early phosphorylation events and alter intracellular pH. These ions activate transporters, channel proteins, membrane-bound receptor kinases, and some TFs within minutes, then affect biological processes such as specialized metabolism [29,30]. We also found that changes in soil nitrogen or moisture levels led to the induction of distinct metabolic pathways. Increasing nitrogen levels under drought conditions can promote the expression of genes encoding photopigment complex proteins, electron, and proton transporter proteins, and oxygen release complex proteins, which in turn increase light energy capture, conversion efficiency, and water photolysis efficiency. Multiple rate-limiting enzyme-encoding genes in the CBB cycle were up-regulated, potentially enhancing carbon assimilation efficiency. This also increased the saturated vapor pressure difference within leaf pulp cells and water utilization efficiency in leaves. Further analysis of DEGs related to tuber expansion, we hypothesize that expression of *StaGL8*, *PATATIN*, *StPHYB2*, and other regulatory genes in leaves in response to sufficient nitrogen levels resulted in down-regulation of *StSP6A*, which inhibited stolon expansion and prolonged the vegetative growth period. Transcriptome profiles at multiple time points after ammonia treatment show that increased ammonia levels significantly induce genes related to transcriptional regulation, plant defense and immunity, and JA responses in the short term [31]. In the present study, increased water and nitrogen levels induced the expression of hormone-related genes, such as those in the auxin, gibberellin, and ethylene pathways. In our study, the ethylene-responsive gene (PGSC0003DMG400000066) was identified as differentially expressed in response to changes in both water and nitrogen by crossover analysis of DEGs. Therefore, we hypothesize that water and nitrogen may interact through hormone signaling pathways. Nutrient and hormone signals often interact with each other during plant development; ammonium, as a signaling molecule, can also interact with hormone pathways [32]. Nitrogen is known to regulate the expression of genes in the ABA, auxin, brassinolide, and JA pathways, in addition to others, ultimately affecting plant phenotypes. The auxin transporter *NPF6.3* and the plastid metalloproteinase *AMOS1/EGY1* (a member of the ABA signal transduction pathway) are key interaction genes [33,34,35]. Therefore, we constructed a model of potato responses to changes in water and nitrogen signals at the transcriptional level by integrating components identified as involved in nitrogen absorption, transport, and signal transduction in potato root and leaf tissues under different water and nitrogen treatments (Figure 6).

Many transgenic experiments have shown that manipulating the expression of genes involved in nitrogen transport, assimilation, and signaling can promote crop growth or yield improvement [36]. WGCNA is currently the preferred algorithm for calculating the correlation between genes and phenotypes. Compared with the bioinformatics method that only analyzed the differentially expressed genes, our work required a high-power computer and carefully distinguished the false-positive results [37]. Here, 34 key candidate genes that responded to changes in water and nitrogen levels were identified via WGCNA. Several of the candidates, including *MYB*, *DOF*, an amino acid transporter, and aquaporin, have been reported to play important roles in nitrogen uptake and utilization [38,39,40,41]. In conclusion, the key genes identified in this study are promising candidate molecular targets for future improvement of water and fertilizer use efficiency in potatoes.

After the external application of urea, substances such as NH_4_^+^, NO_3_^−^, and CO_3_^2−^ are generated by the actions of soil microorganisms and water. NH_4_^+^ enters a root cell through an ammonia transporter, assisted by a proton pump. NO_3_^−^ enters the cell through nitrate transporters. Urea and water enter the cell through aquaporins. After absorbance into cells through membrane transporters, some compounds are assimilated and catalyzed by nitrate reductase and glutamate dehydrogenase; the remainder are transported to leaf cells for absorption and assimilation by transporters. Entry of NH_4_^+^ and NO_3_^−^ into a cell alters intracellular pH and Ca^2+^ levels, which causes responses by calmodulin (CaM), calcium-dependent protein kinase (CDPK), reactive oxygen species (ROS), and other signaling molecules. Various reaction cascades ultimately affect the plant’s morphological phenotype. 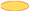 are differentially expressed genes (DEGs) in response to changes in both nitrogen and water; 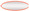 are DEGs in response to changes in either water or nitrogen but not both. Dashed lines separate changes induced by different treatments.

## 4. Conclusions

In this study, photosynthetic characteristics, biomass, yield, and other physiological indexes were measured in potato plants in response to four water and nitrogen treatments. Furthermore, transcriptomic analysis was conducted to identify differentially expressed genes in metabolic pathways in roots and leaves. Nitrogen limitation was found to accelerate stolon terminal expansion, whereas sufficient nitrogen levels prolonged the stolon growing period. Under drought conditions, increasing nitrogen levels decreased the leaf stomatal conductance but increased intracellular saturated vapor pressure difference, water photolysis efficiency, light energy conversion efficiency, and carbon assimilation efficiency. This had no significant effect on yield but increased tuber protein content. Under sufficient nitrogen conditions, increasing water levels decreased the relative chlorophyll content and saturated water vapor pressure difference but increased the net photosynthetic rate and yield. A total of 34 key candidate genes that respond to changes in soil water and nitrogen content were identified, and a preliminary molecular model of potato responses to alterations in soil water and nitrogen content was constructed. These results serve as a theoretical reference for water and fertilizer management in potatoes and provide a set of promising target genes for molecular improvement of water and fertilizer use efficiency in potatoes.

## Figures and Tables

**Table 1:** Relative water content and amounts of nitrogen applied for potato treatment groups.

Treatment	Level	Seedling Stage	Tuber Formation Stage	Tuber Expansion Stage	Starch Accumulation Stage
Urea/(g)	Limited (N1)	2.24	0	0	0
	Sufficient (N3)	7.78	2.22	1.11	0
Moisture content/(%)	Deficit (W1)	35~45	45~55	50~60	30~40
	Adequate (W3)	75~85	85~95	90~100	70~80

## Data Availability

The data used in this study were deposited at the National Center for Biotechnology Information (NCBI) under the project name PRJNA863742.

## Supplementary Materials

The following supporting information can be downloaded at: https://www.mdpi.com/article/10.3390/plants12081671/s1, Figure S1: Validation of DEGs by RT-qPCR; Figure S2: GO enrichment of DEGs; Figure S3: KEGG enrichment of DEGs; Figure S4: Protein content of tuber; Figure S5: KEGG enrichment analysis for the blue and turquoise modules; Figure S6: Blue and Turquoise module protein interaction network; Table S1: Primers used to quantify the relative expression of genes via RT-qPCR; Table S2: Quality of the transcriptome sequencing data; Table S3: Unique and shared DEGs between treatment groups in leaf tissue; Table S4: Unique and shared DEGs between treatment groups in root tissue; Table S5: Photosynthetic-related gene expression after nitrogen application; Table S6: Stress-related gene expression after nitrogen application; Table S7: Nitrogen metabolism-related pathway gene expression; Table S8: Tuber developing-related genes expression; Table S9: The 34 candidate key genes responding to changes in water and nitrogen levels.
