# Supplementary material for: Analysis of Potato Physiological and Molecular Adaptation in Response to Different Water and Nitrogen Combined Regimes
Source: Plants (Basel). 2023 Apr 17;12(8):1671. doi: 10.3390/plants12081671 (PMC10145361; doi:10.3390/plants12081671)
Supplement: Supplementary file 1 [file plants-12-01671-s001.zip › Supplementary Figure.pdf]

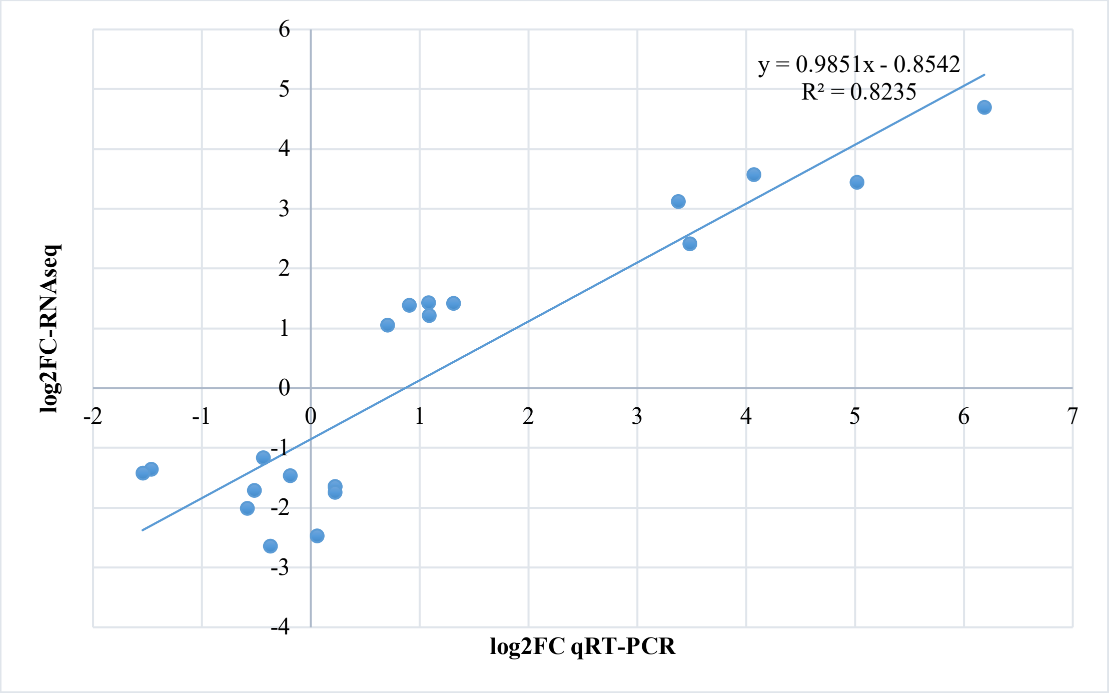

Figure S1 Validation of DEGs by RT-qPCR

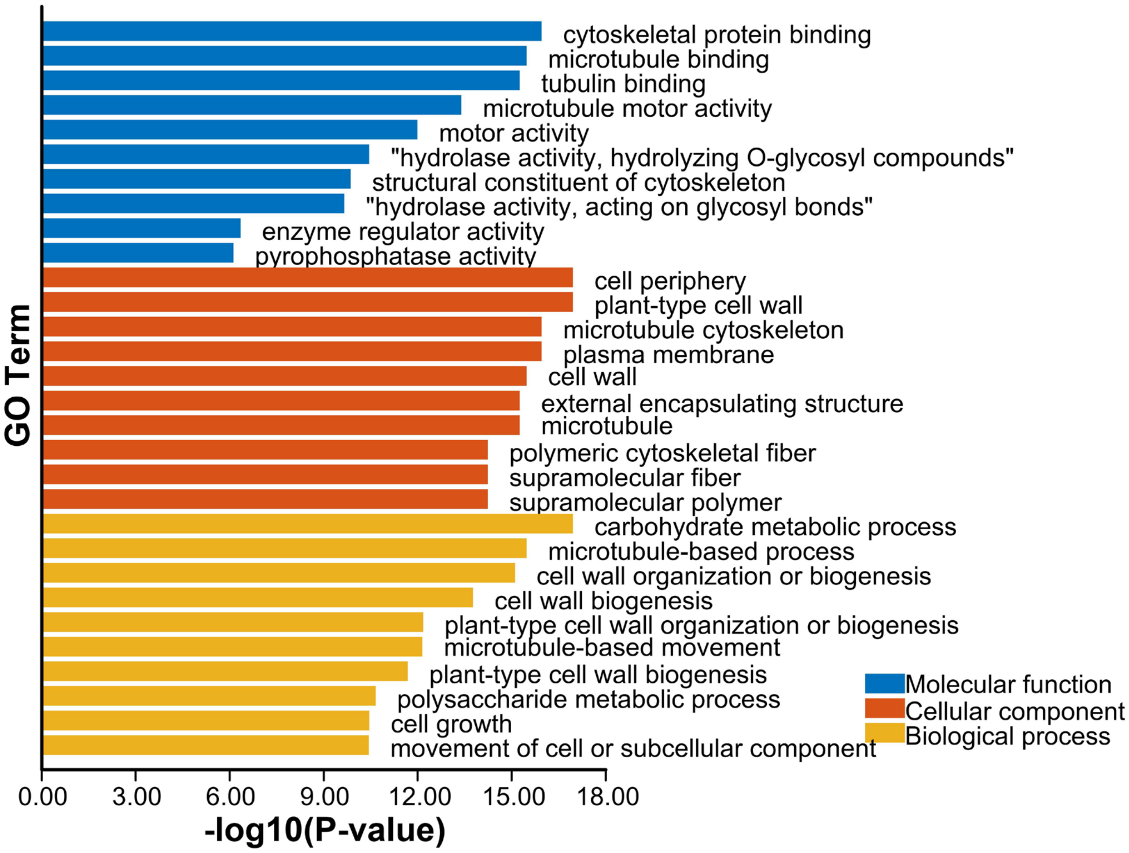

a. DEGS of W1N1 VS W1N3 in Root

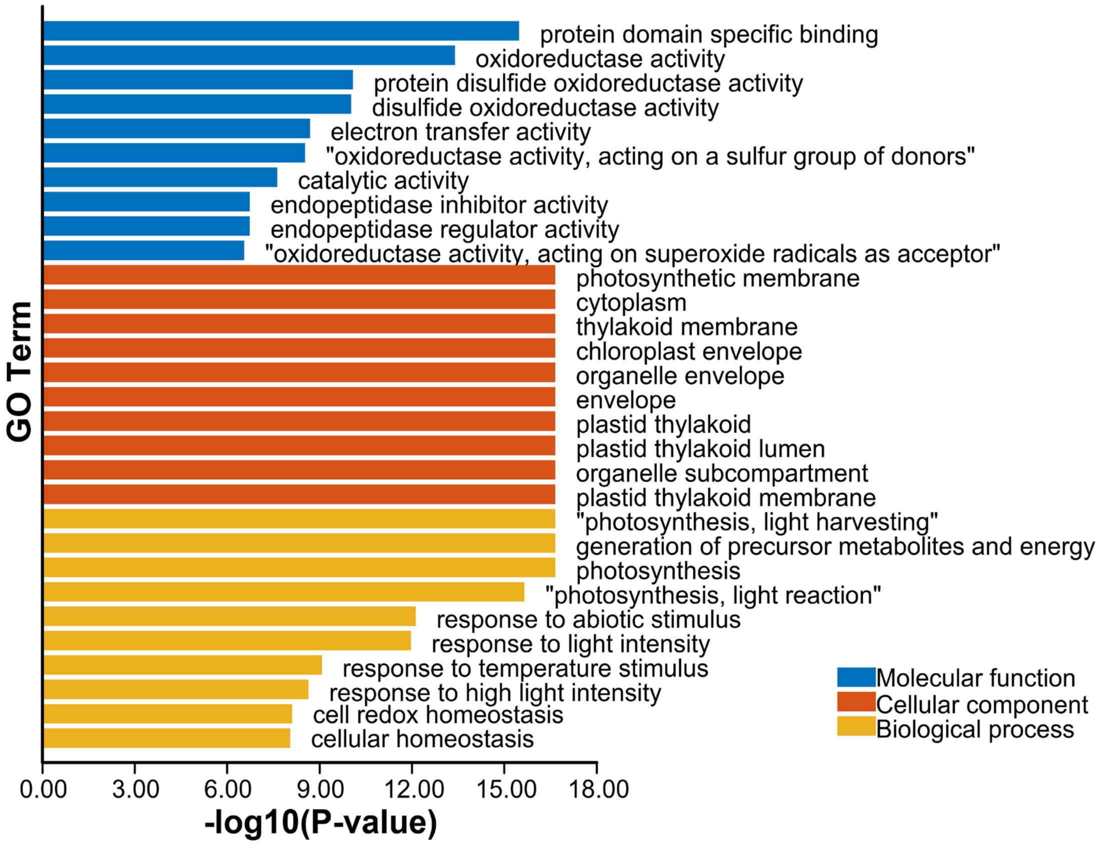

b. DEGS of W1N1 VS W1N3 in Leaf

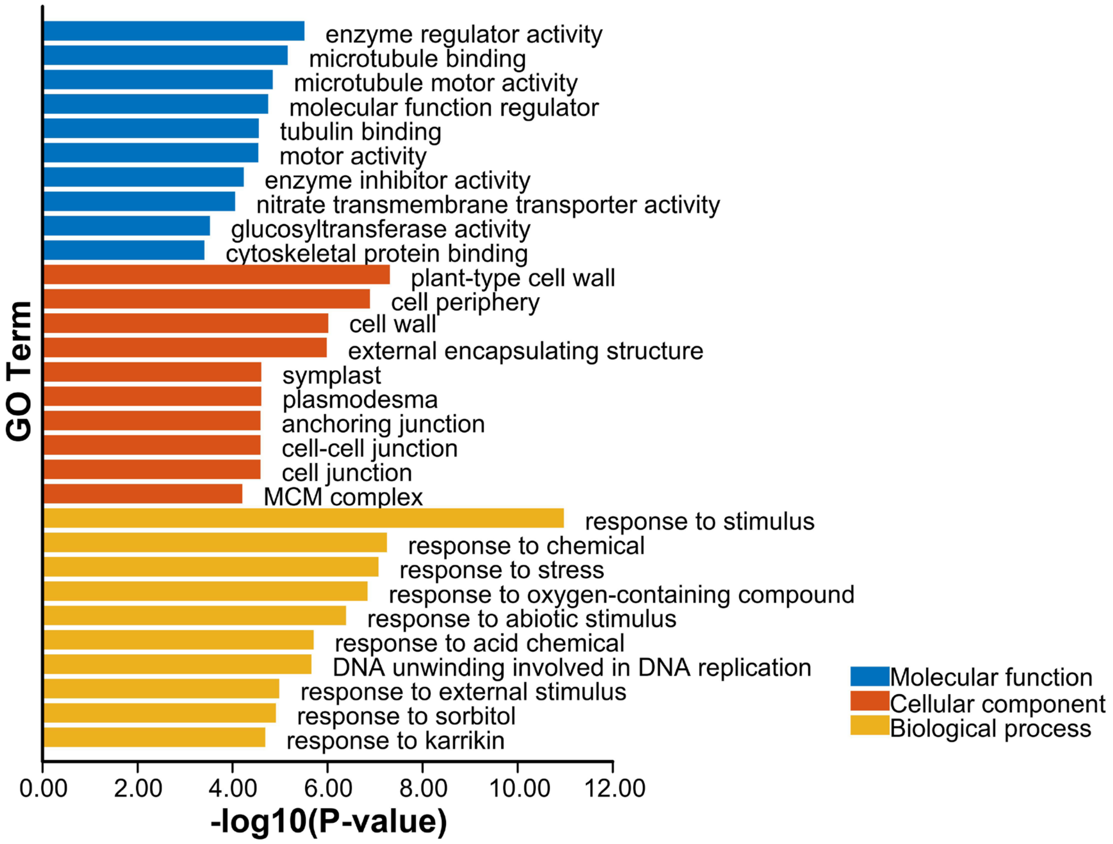

c. DEGS of W3N1 VS W3N3 in Root

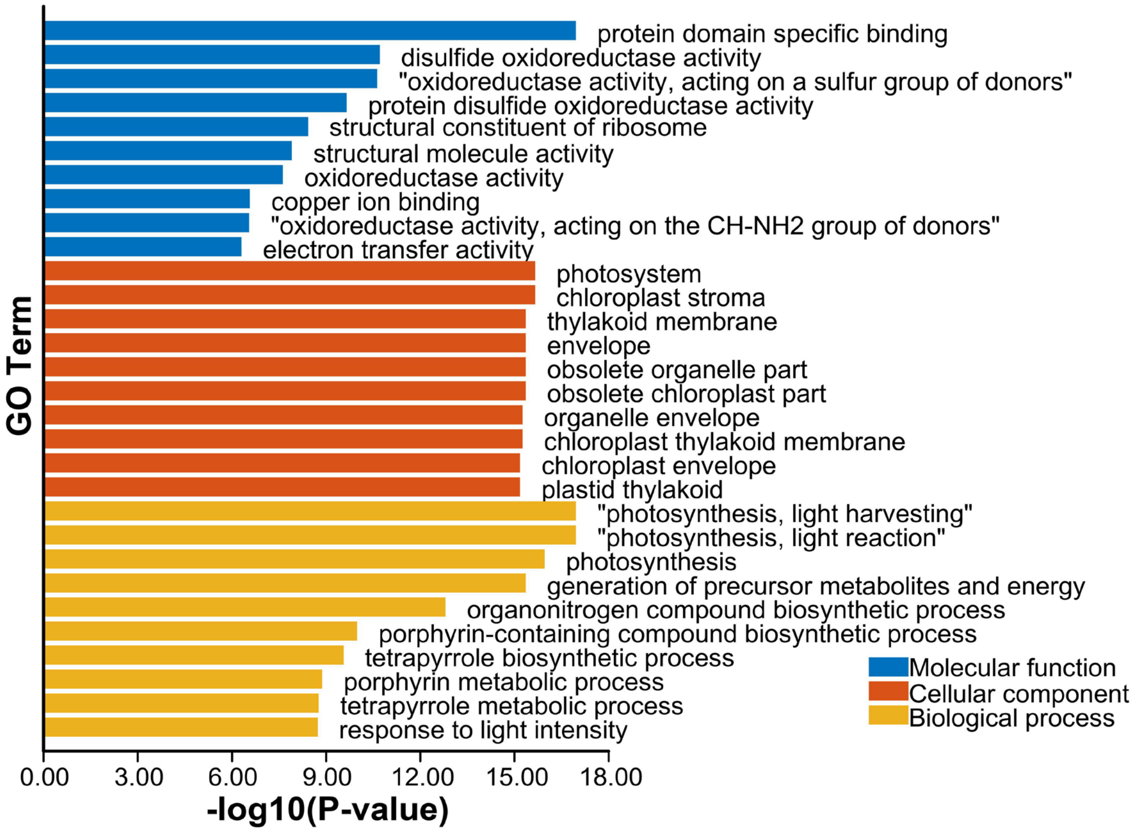

d. DEGS of W3N1 VS W3N3 in Leaf

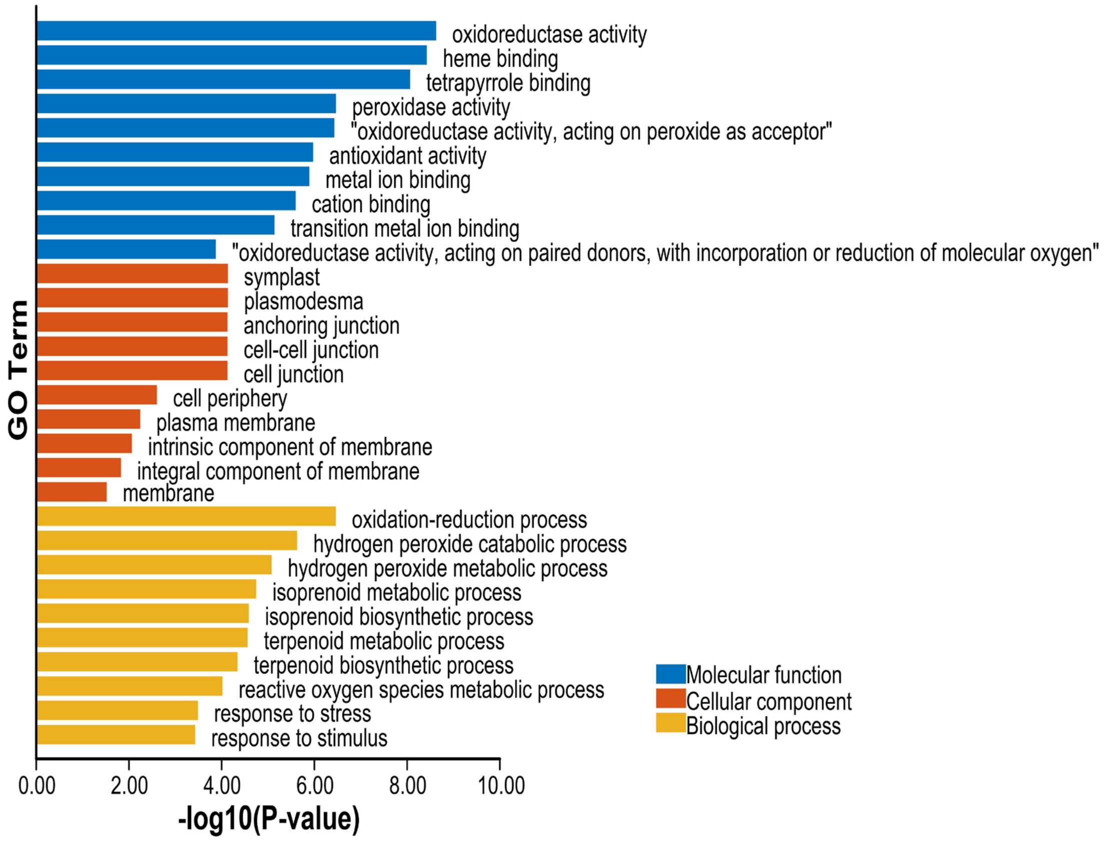

e. DEGS of W1N1 VS W3N1 in Root

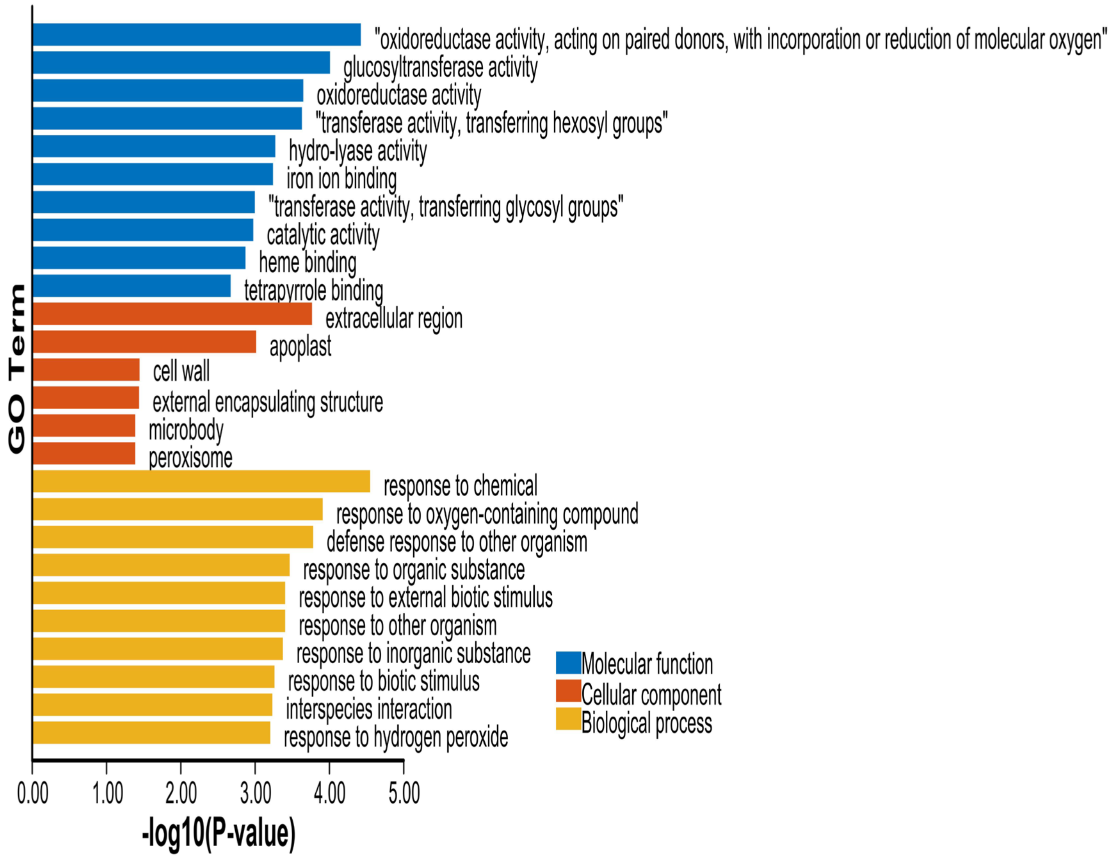

f. DEGS of W1N1 VS W3N1 in Leaf

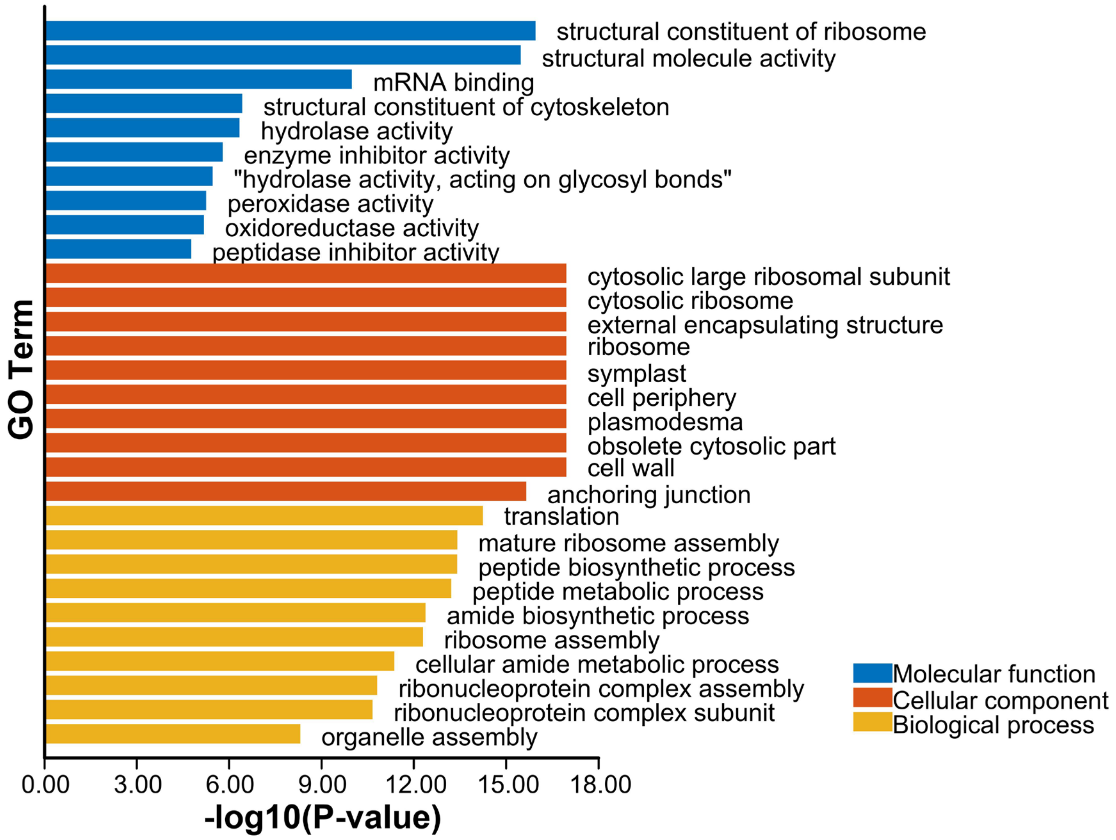

g. DEGS of W1N3 VS W3N3 in Root

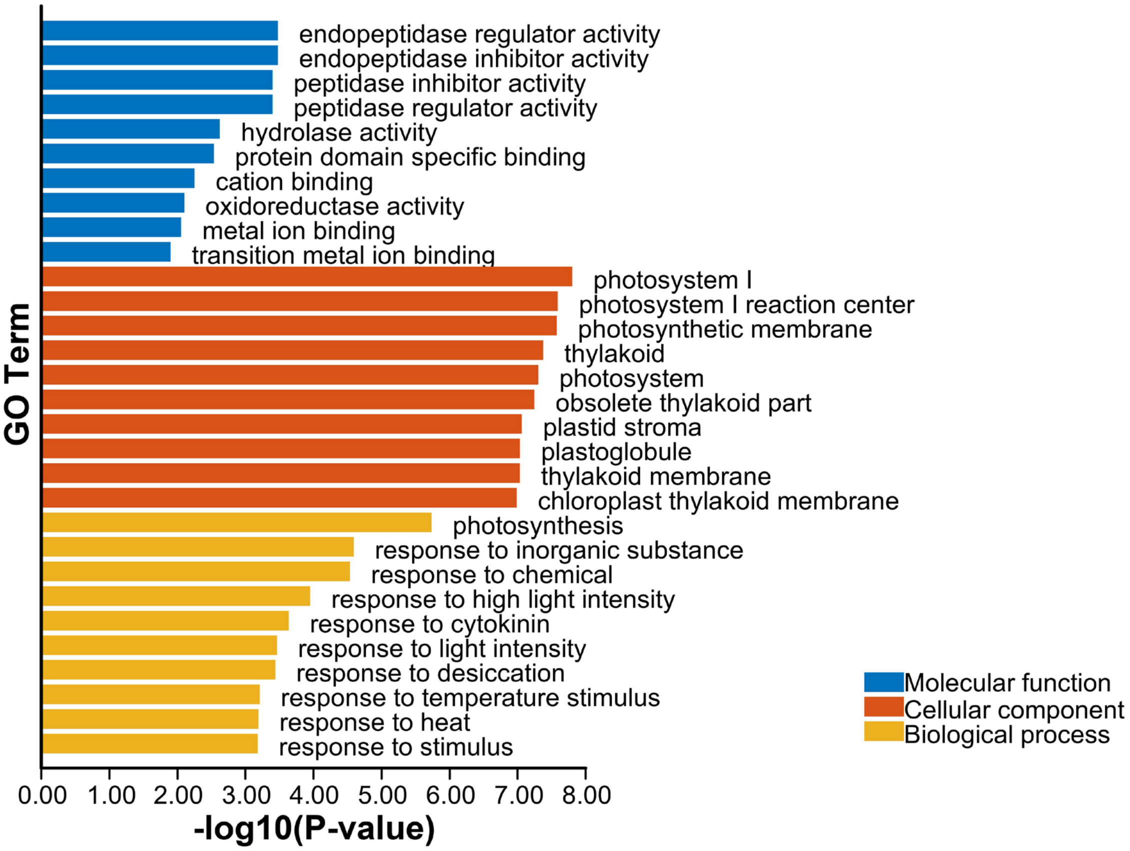

h. DEGS of W1N3 VS W3N3 in Leaf

Figure S2 GO enrichment of DEGs

Note: The top ten terms of MF, CC and BP are selected for display in different treatment groups.

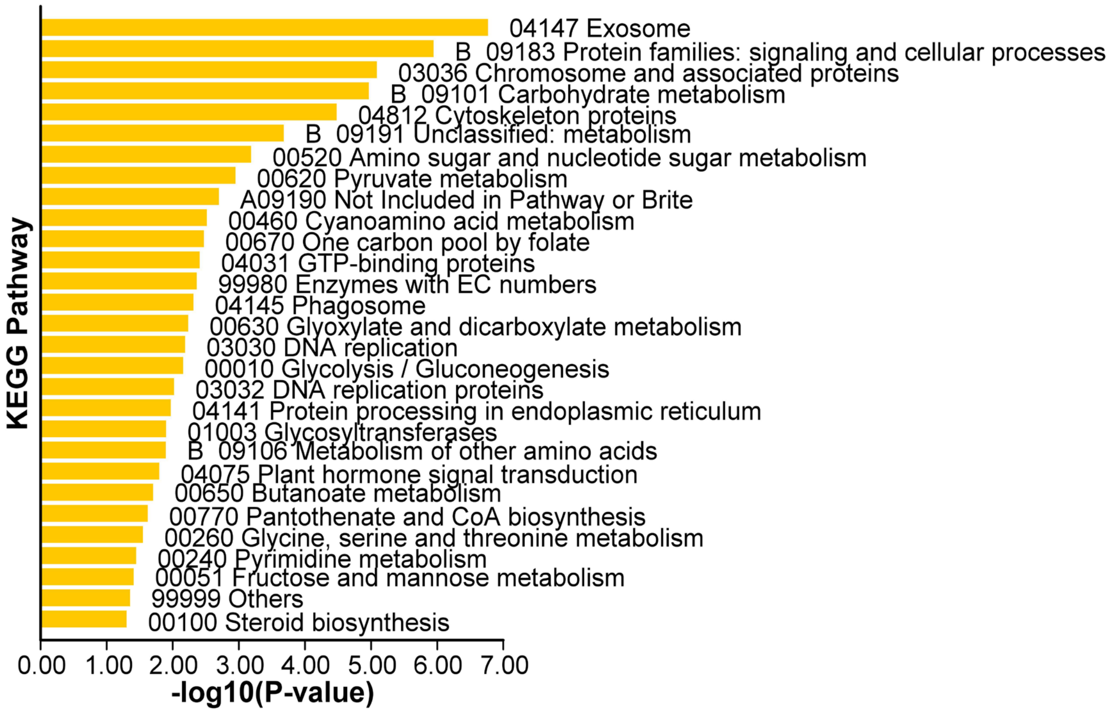

a. DEGS of W1N1 VS W1N3 in Root

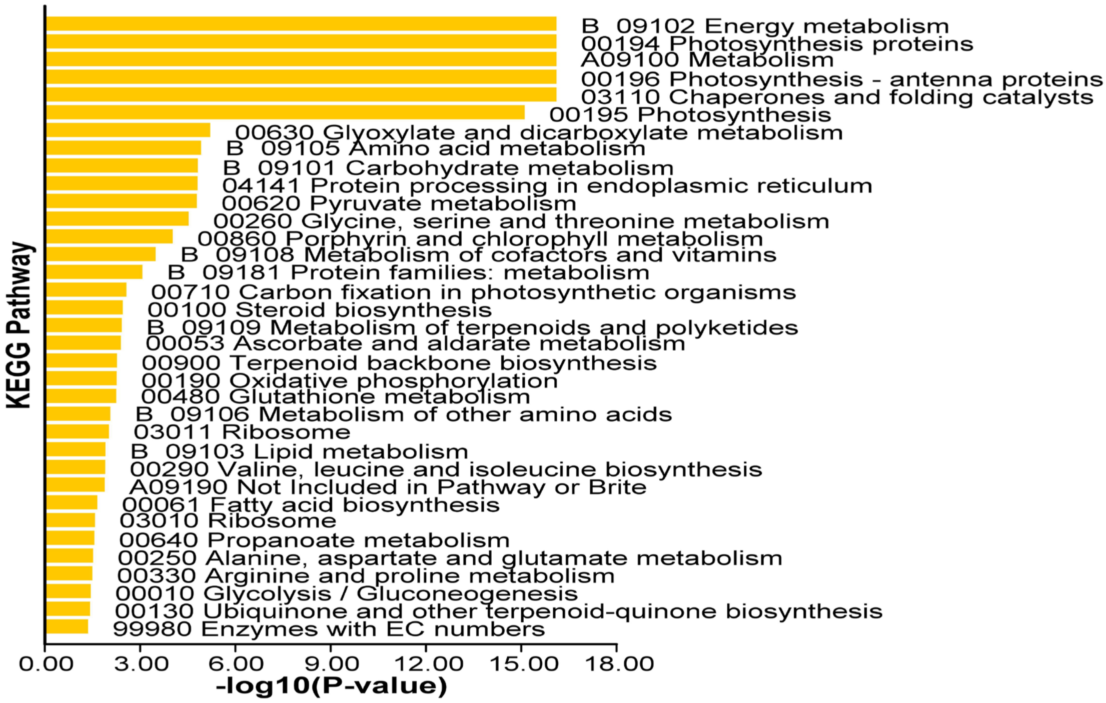

b. DEGS of W1N1 VS W1N3 in Leaf

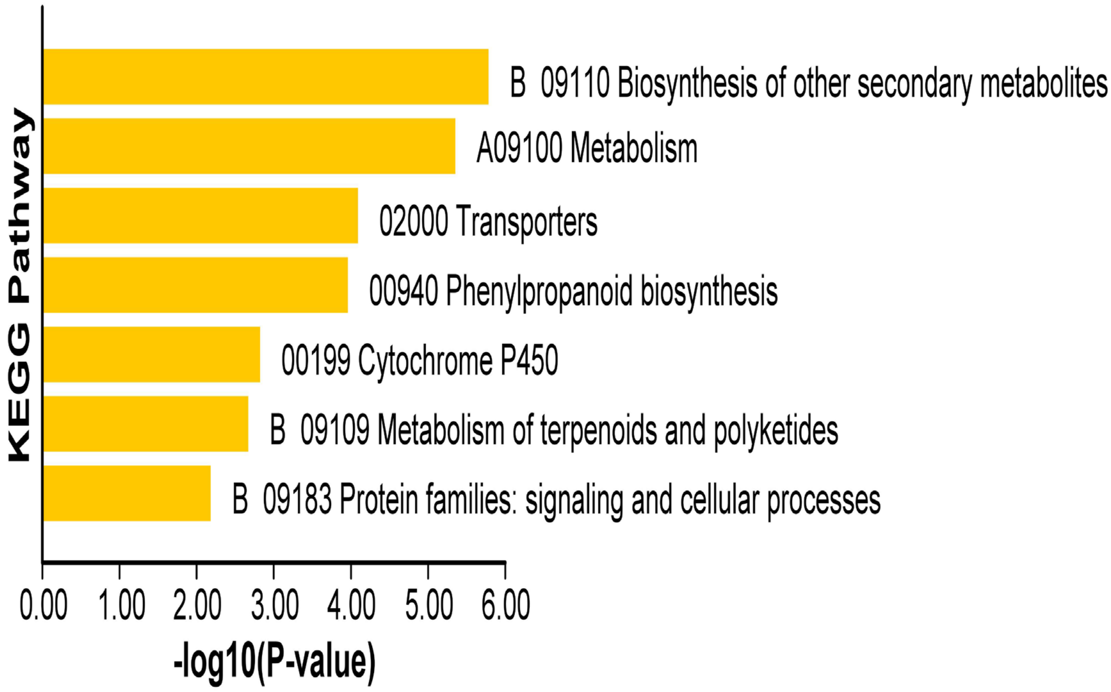

c. DEGS of W1N1 VS W3N1 in Root

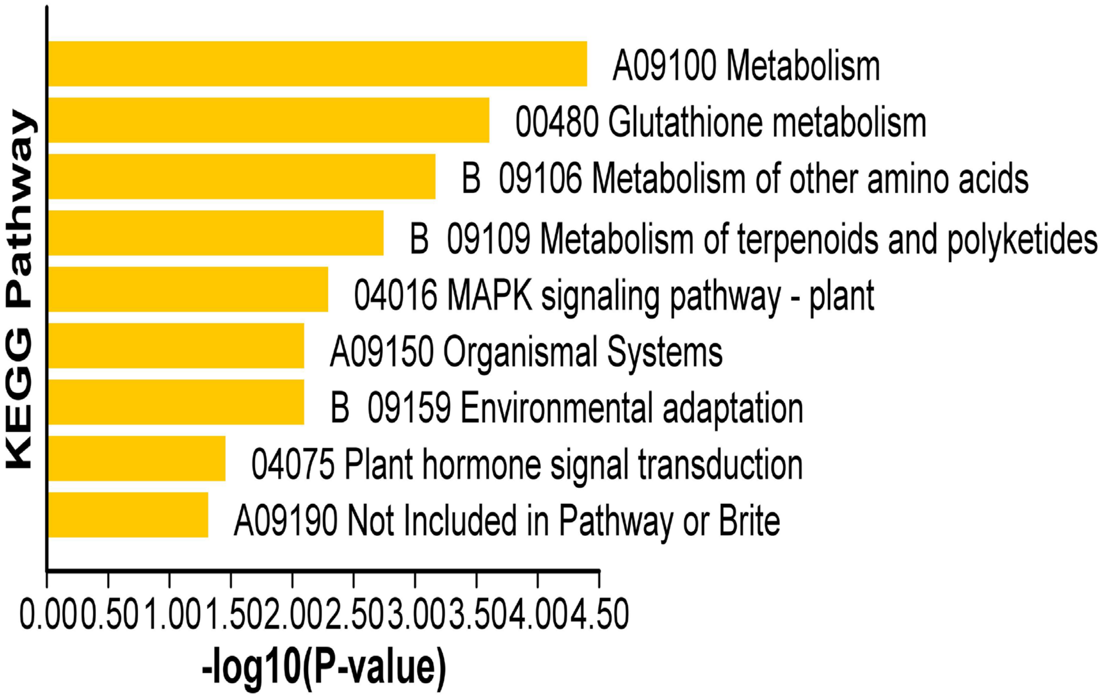

d. DEGS of W1N1 VS W3N1 in Leaf

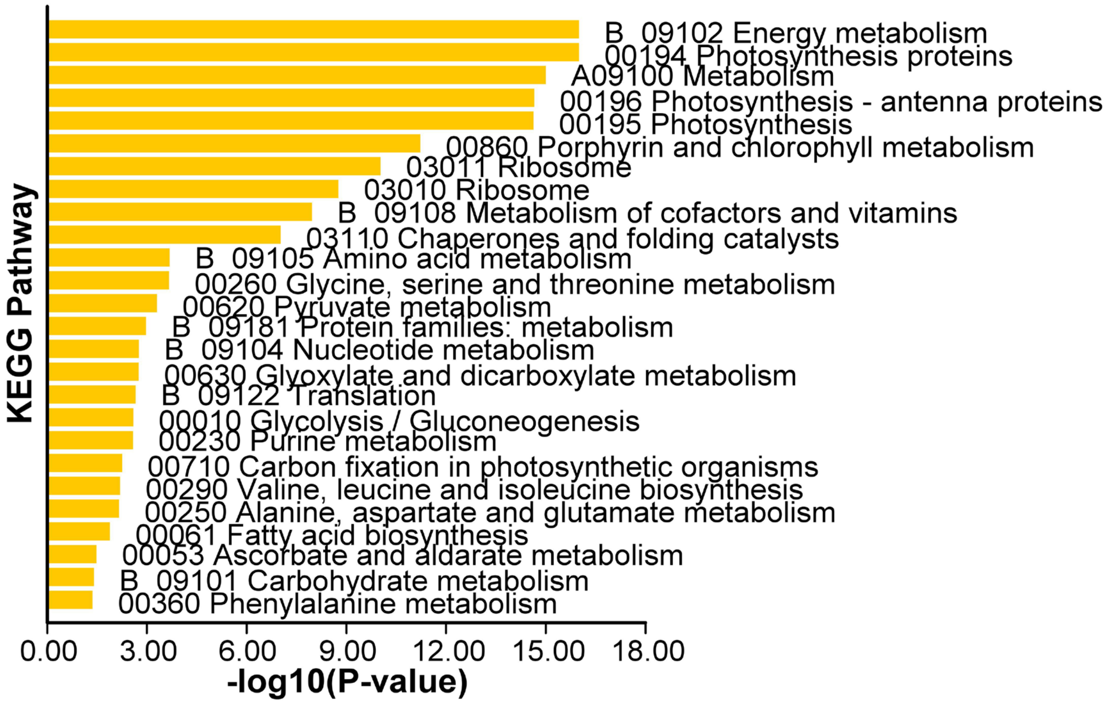

e. DEGS of W3N1 VS W3N3 in Root

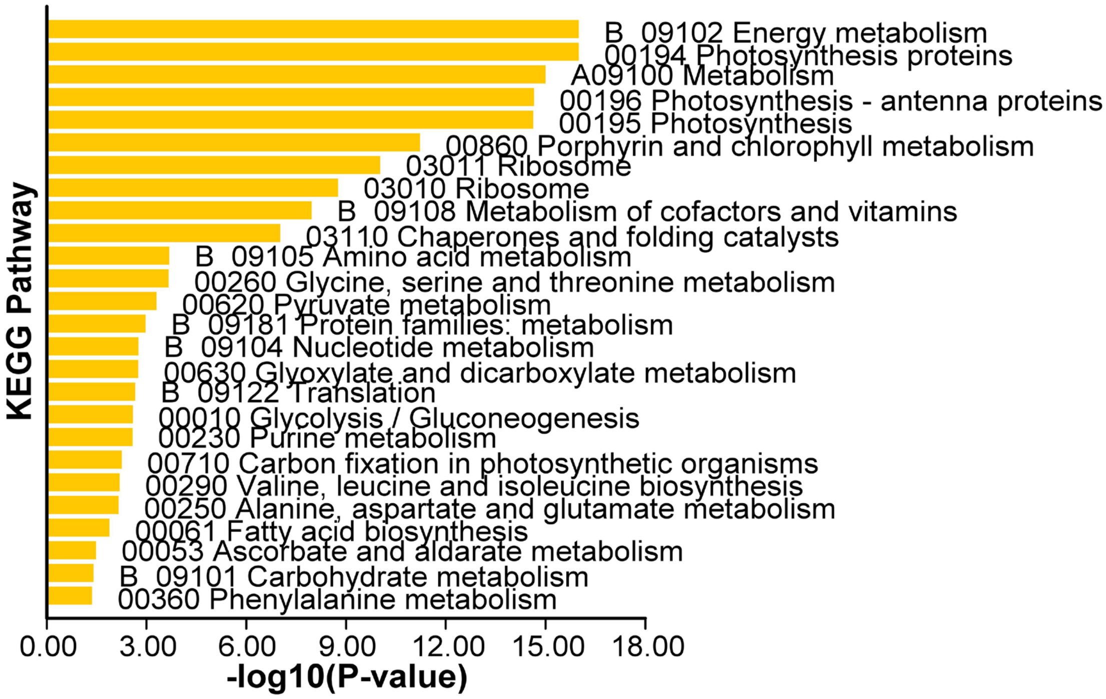

f. DEGS of W3N1 VS W3N3 in Leaf

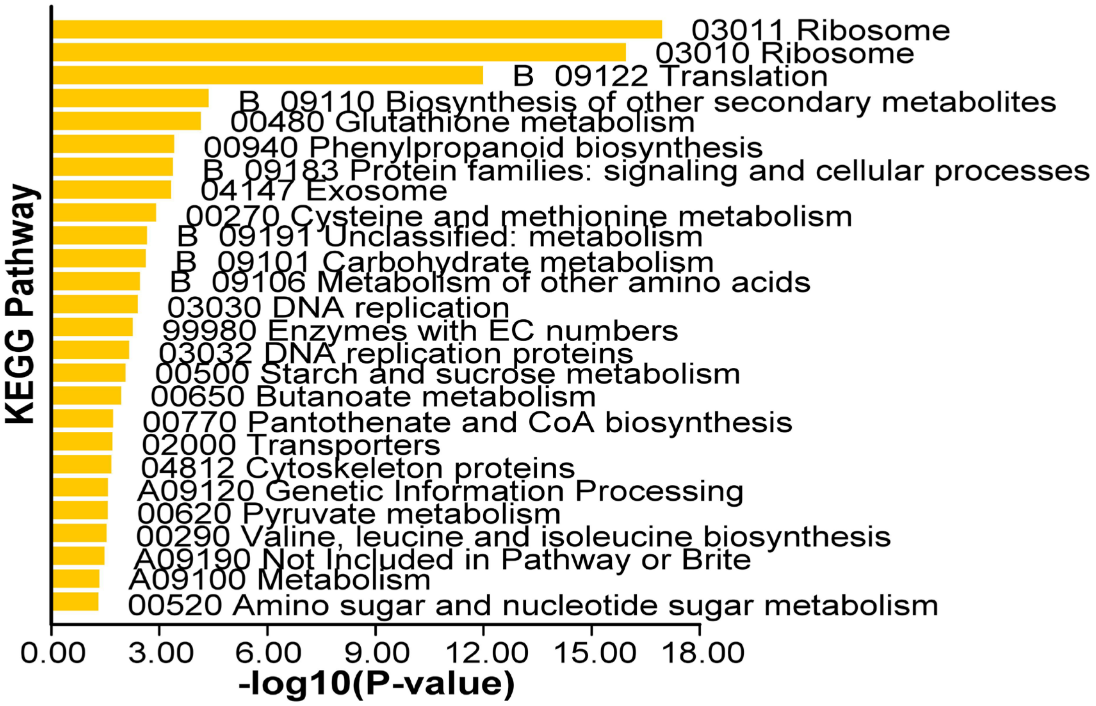

g. DEGS of W1N3 VS W3N3 in Root

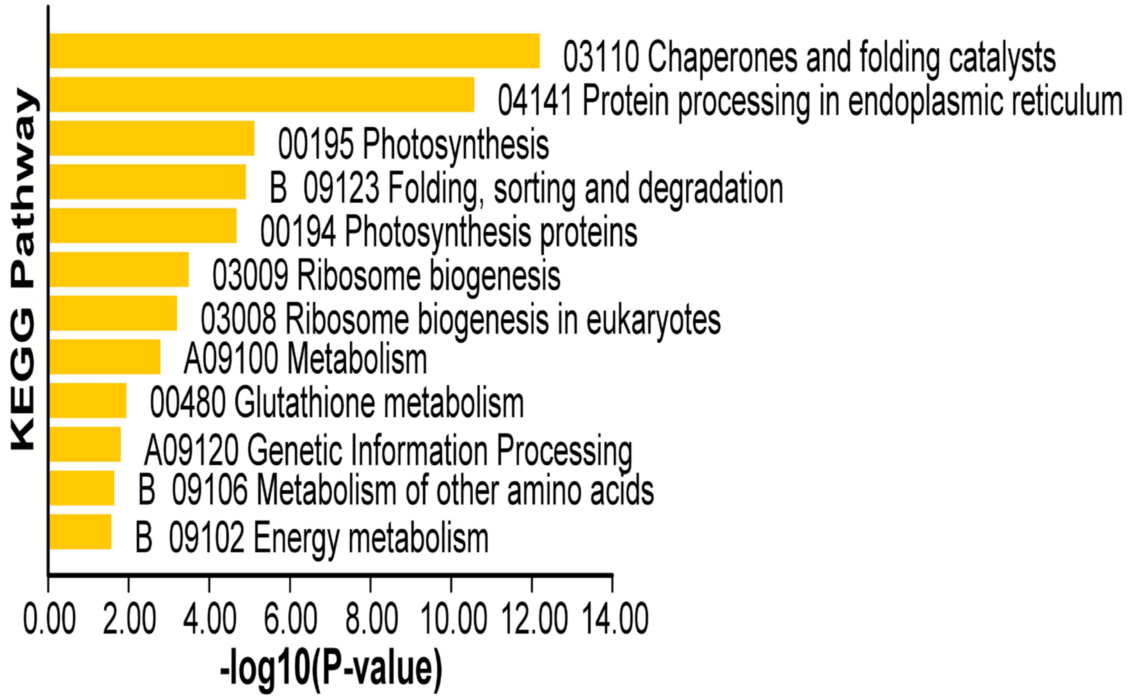

h. DEGS of W1N3 VS W3N3 in Leaf

Figure S3 KEGG enrichment of DEGs

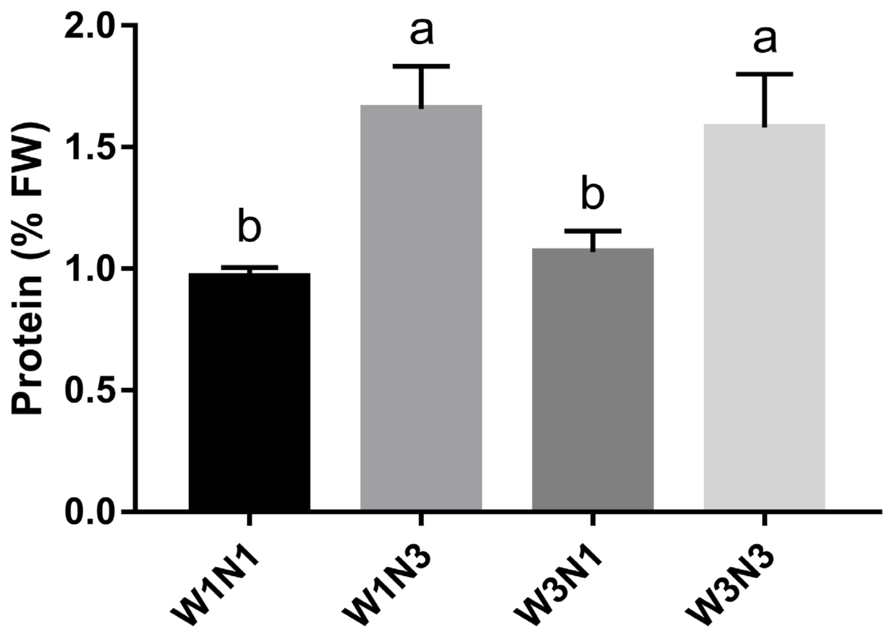

Figure S4 Protein content of tuber

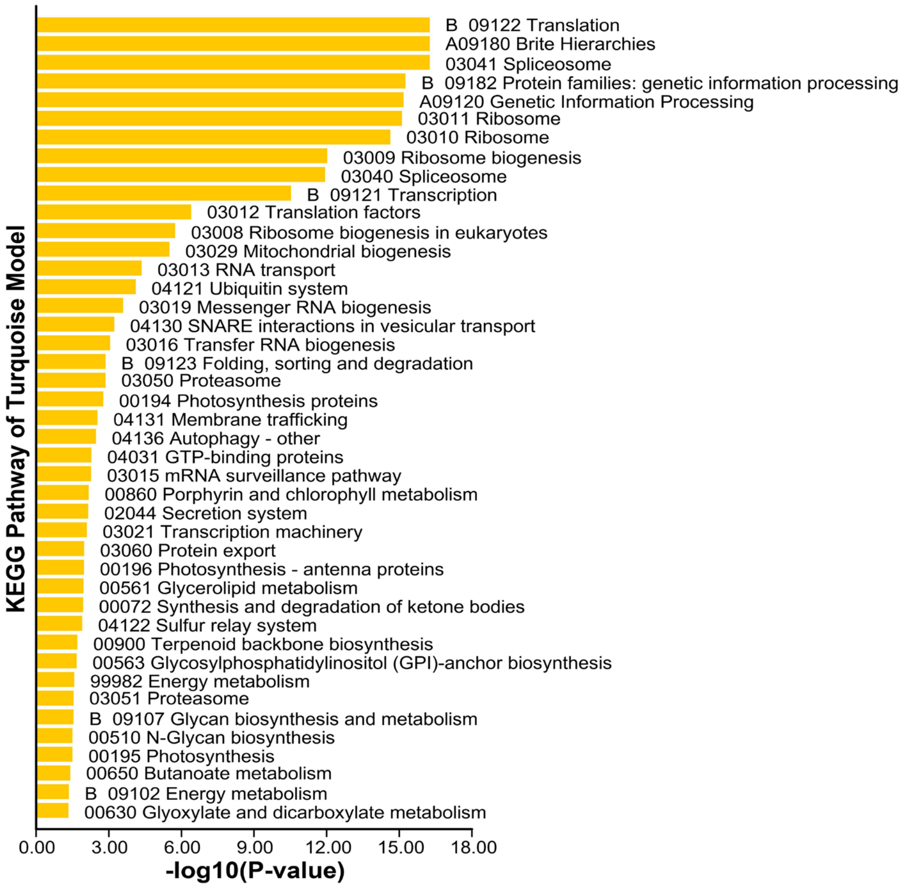

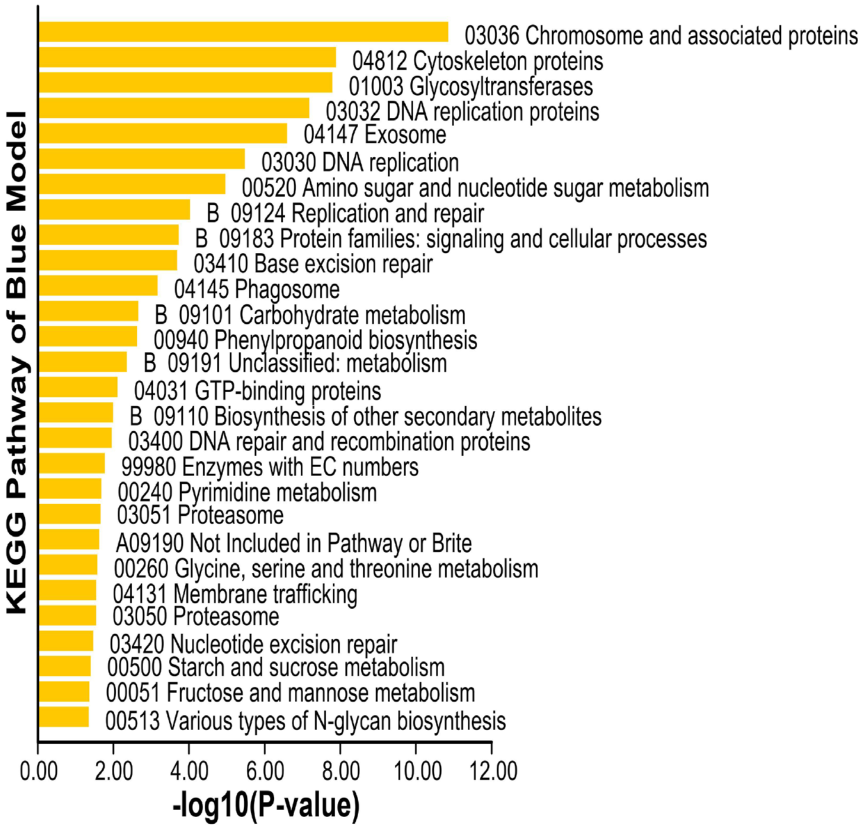

Figure S5 KEGG enrichment analysis for the blue and turquoise modules

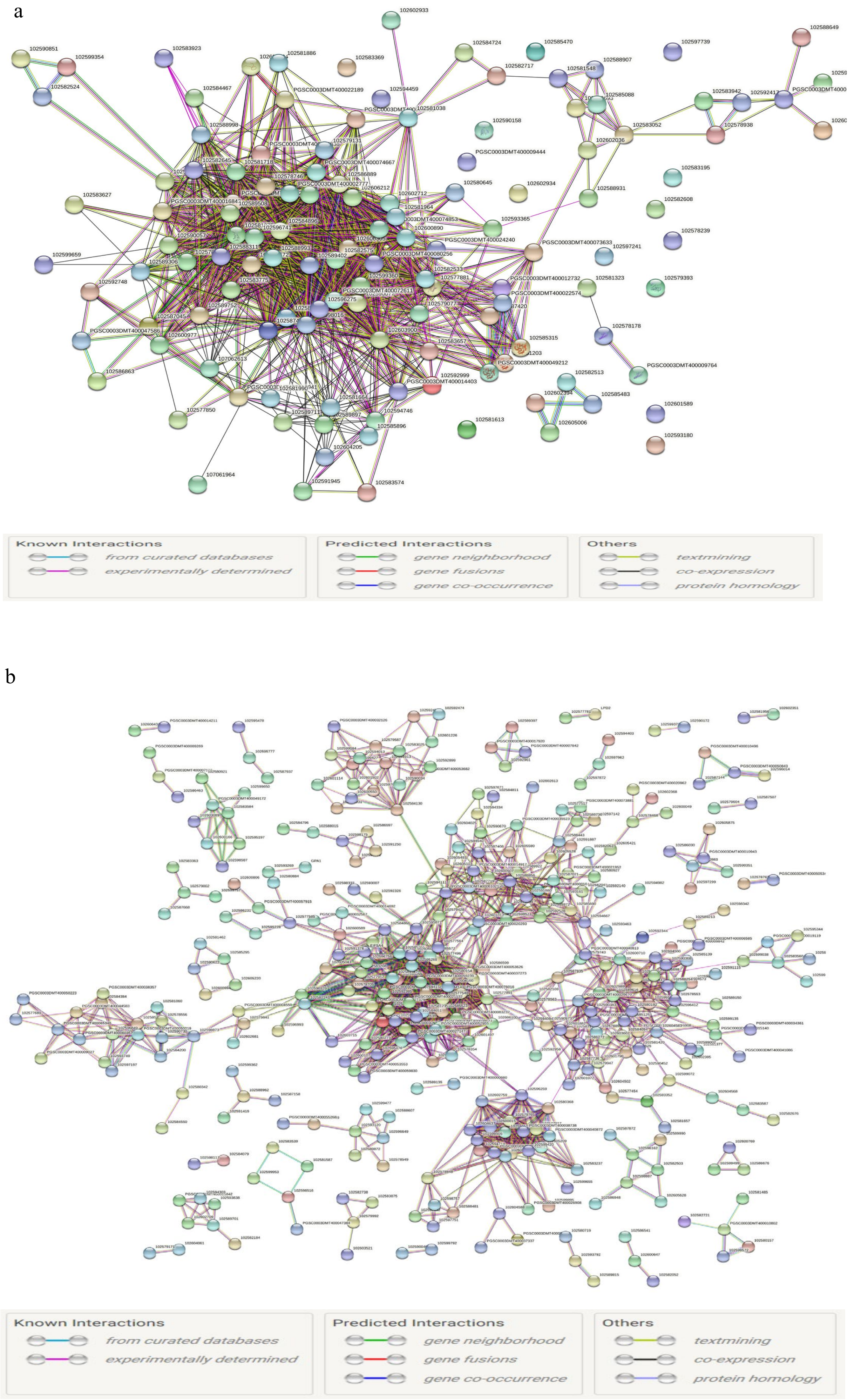

Figure S6 Blue (a) and Turquoise (b) module protein interaction network
